# Supplementary material for: Dnmt3a is an epigenetic mediator of adipose insulin resistance
Source: eLife. 2017 Nov 1;6:e30766. doi: 10.7554/eLife.30766 (PMC5730374; doi:10.7554/eLife.30766)
Supplement: Supplementary file 3. [file elife-30766-supp3.docx]

**Supplemental Table 3. Oligonucleotide sequences used in this manuscript**

| Hairpin | shDnmt1 #1 | ACCAAGCTGTGTAGTACTTTG |
| --- | --- | --- |
| Hairpin | shDnmt1 #2 | TATATGAAGACCTGATCAATA |
| Hairpin | shDnm3a #1 | CGCTCCGCTGAAGGAATATTT |
| Hairpin | shDnm3a #2 | CCAGATGTTCTTTGCCAATAA |
| Hairpin | shDnmt3b #1 | GCACTTTAATCTGGCTACCTT |
| Hairpin | shDnmt3b #2 | CAAGAAAGACATCTCAAGATT |
| Q-PCR | Cyclophilin_F | GGTGGAGAGCACCAAGACAGA |
| Q-PCR | Cyclophilin_R | GCCGGAAGTCGACAATGATG |
| Q-PCR | Dnmt1_f | GAGGACTGCAACGTGCTTCT |
| Q-PCR | Dnmt1_r | GAGCGTCTGTAGGACACGAA |
| Q-PCR | Dnmt3a_f | GTGGAGCCTGAAGCAGCTG |
| Q-PCR | Dnmt3a_r | CTGGCACATGCCTCCAATGAA |
| Q-PCR | Dnmt3b_f | CCATGGTGGTGTCCTGGAAA |
| Q-PCR | Dnmt3b_r | CAGGACTGCTGGAGAAGGTCT |
| Q-PCR | Fgf21_f | TCCAAATCCTGGGTGTCAAA |
| Q-PCR | Fgf21_r | CAGCAGCAGTTCTCTGAAGC |
| Q-PCR | Ucp1_f | CACCTTCCCGCTGGACACT |
| Q-PCR | Ucp1_r | CCCTAGGACACCTTTATACCTAATGG |
| Q-PCR | Cidea_f | ATCACAACTGGCCTGGTTACG |
| Q-PCR | Cidea_r | TACTACCCGGTGTCCATTTCT |
| Q-PCR | Elovl3_f | TCCGCGTTCTCATGTAGGTCT |
| Q-PCR | Elovl3_r | GGACCTGATGCAACCCTATGA |
| Q-PCR | Dio2_f | CAGTGTGGTGCACGTCTCCAATC |
| Q-PCR | Dio2_r | TGAACCAAAGTTGACCACCAG |
| Q-PCR | Ppargc1a_f | AGCCGTGACCACTGACAACGAG |
| Q-PCR | Ppargc1a_r | GCTGCATGGTTCTGAGTGCTAAG |
| Q-PCR | Ccl2_f | GTCCCTGTCATGCTTCTGG |
| Q-PCR | Ccl2_R | GCGTTAACTGCATCTGGCT |
| Q-PCR | Tnfa_f | ATGAGAAGTTCCCAAATGGC |
| Q-PCR | Tnfa_r | CTCCACTTGGTGGTTTGCTA |
| Q-PCR | Il6_f | CTCTGGGAAATCGTGGAAAT |
| Q-PCR | Il6_r | CCAGTTTGGTAGCATCCATC |
| Q-PCR | F4/80_f | CTTTGGCTATGGGCTTCCAGTC |
| Q-PCR | F4/80_r | GCAAGGAGGACAGAGTTTATCGTG |
| MeDIP-qPCR | Fgf21- #1-f | CCAAACCCAGGGGGCCCATGCCT |
| MeDIP-qPCR | Fgf21- #1-r | CTGTCTGGGTATAAATTCTGGTA |
| MeDIP-qPCR | Fgf21-#2-f | TCTGAAGTGAGGCTGGAGACAAA |
| MeDIP-qPCR | Fgf21-#2-r | TCCCAAGGAGCATCTAAGGGGA |
| MeDIP-qPCR | Fgf21-#3-f | TGAAACTGACTGAAGGCTCAGAGAC |
| MeDIP-qPCR | Fgf21-#3-r | AGAGAACCCAGAAGGGACAGGGGC |
| MeDIP-qPCR | Fgf21-#4-f | AGGGTCCAGCACTGGCAGGGAGATT |
| MeDIP-qPCR | Fgf21-#4-r | CAGGAGGCAGGTTTGGTAAC |
| MeDIP-qPCR | Fgf21-distal1-f | GTTCTTCTGTATGCAAGCC |
| MeDIP-qPCR | Fgf21-distal1-r | TAGACCAGGCTGTTCTCAAAC |
| MeDIP-qPCR | Fgf21-distal2-f | GTGGTGGCTTCATAGAGAGAA |
| MeDIP-qPCR | Fgf21-distal2-r | TAAAGGCAAATGGGACAAAGGGAA |
| MeDIP-qPCR | Fgf21-distal3-f | GGGAGACCCAGGTGTGGCAGT |
| MeDIP-qPCR | Fgf21-distal3-r | GTTTCACATGTTTGCCCCAGC |
