# Supplementary material for: Dnmt3a is an epigenetic mediator of adipose insulin resistance
Source: eLife. 2017 Nov 1;6:e30766. doi: 10.7554/eLife.30766 (PMC5730374; doi:10.7554/eLife.30766)
Supplement: Supplementary file 4. [file elife-30766-supp4.docx]

**Supplemental Table 4.** Clinical characteristics of study subjects included in the discordant twin cohort and the case control cohort. These clinical data have previously been published (Nilsson et al., 2014).

|  | **Discordant twins** | | **Case-control cohort** | |
| --- | --- | --- | --- | --- |
|  | Non-diabetic | T2D^a^ | NGT | T2D |
| N (male/female) | 14 (9/5) | 14 (9/5) | 28 (15/13) | 28 (15/13) |
| Age (years) | 67.6 ± 7.7 | 67.6 ± 7.7 | 74.3 ± 4.3 | 74.5± 4.2 |
| BMI (kg/m^2^) | 29.8 ± 6.8 | 32.0 ± 7.1* | 27.0 ± 3.6 | 27.4 ± 3.6 |
| Fasting plasma glucose (mmol/l) | 6.0 ± 0.5 | 9.3 ± 2.6^#^ | 5.5 ± 0.5 | 7.1 ± 1.9^#^ |
| 2-hour glucose (mmol/l) | 8.3 ± 1.8 | 16.1 ± 5.2* | 6.6 ± 0.7 | 15.1 ± 5.0^#^ |
| HbA1c (mmol/mol) | 41.0 ± 4.4 | 58.0 ± 19.7* | 39.0 ± 3.3 | 49.0 ± 14.0^#^ |

Data are shown as mean ± SD. Among the discordant twins, the non-diabetic co-twin had normal glucose tolerance (NGT) in 4 pairs and impaired glucose tolerance (IGT) in 10 pairs. Glucose tolerance was determined according to the 1999 World Health Organization criteria. T2D; type 2 diabetic, a.u.; arbitrary units. * *p<*0.05 and ^#^ *p<*0.001 between T2D and non-diabetic/NGT subjects. ^a^ In discordant twins, 11% of the T2D subjects received insulin treatment, 33% received oral agents (metformin, sulfonylurea or rosiglitazone) and 33% received both insulin and oral agents.

Nilsson, E., Jansson, P. A., Perfilyev, A., Volkov, P., Pedersen, M., Svensson, M. K., et al. (2014). Altered DNA methylation and differential expression of genes influencing metabolism and inflammation in adipose tissue from subjects with type 2 diabetes. *Diabetes*, *63*(9), 2962–2976. http://doi.org/10.2337/db13-1459
